# Studies on metal charge density and band gap characteristics produced by the (^*n*^BuCp)_2_ZrCl_2_ compound and its reaction mechanism

**DOI:** 10.1039/c8ra01187g

**Published:** 2018-05-21

**Authors:** Hai Wang, Peng Zhang, Panpan Zhou, Renwei Xu, Yu Tang

**Affiliations:** Key Laboratory of Nonferrous Metal Chemistry and Resources Utilization of Gansu Province, State Key Laboratory of Applied Organic Chemistry, College of Chemistry and Chemical Engineering, Lanzhou University Lanzhou 730000 P. R. China tangyu@lzu.edu.cn zhoupp@lzu.edu.cn +86-931-8912582 +86-931-8912552; Lanzhou Petrochemical Research Center, Petrochina Lanzhou 730060 P. R. China

## Abstract

The charge density of the central metal atoms and band gap of the compounds were investigated by the Dmol^3^ software package in Density functional theory (DFT). The band gap of the (^*n*^BuCp)_2_ZrCl_2_ compound was the smallest among the listed metallocene compounds, however, its reactivity reached 11.88 kg (mol h)^−1^ × 10^4^ at the time of slurry polymerization, which was the most active among the catalysts enumerated by the compounds and also verified the Frontier orbital theory. The polymerization of the (^*n*^BuCp)_2_ZrCl_2_ compound with olefins proceeded according to the α-agostic mechanism of the ground state and the transition state. Hydrogen molecules were released during the transition state and led to (^*n*^BuCp)_2_Zr(Cyclopropyl)^+^ as the final product. The authenticity of the presence of hydrogen in the olefin polymerization gas was confirmed. The aggregation of hydrogen led to a decrease in the activity of the metallocene catalyst, and that was why the energy barrier caused by the first polymerization was much lower than the second polymerization. The present work would provide valuable insight into the characteristics of metallocene catalysts with high activity and low hydrogen evolution.

## Abstract 2

The charge density of the central metal atoms and band gap of the compounds were investigated by the Dmol^3^ software package in Density functional theory (DFT).

## Graphical abstract

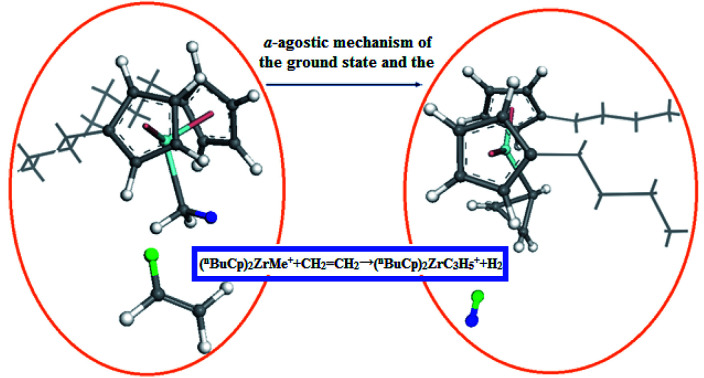

## 1. Introduction

Organometallic molecules are increasingly being used in novel applications because their electronic and magnetic properties can be adjusted by changing the metal elements.^1,2^ As a member of the organometallic compound, metallocenes are composed of a metal atom locating between two planar aromatic ligands. Metallocenes can be generally carried using several immobilization procedures, which have been discussed by Severn and Chadwick.^3^ It has been shown that silica/methylaluminoxane (MAO) cocatalyst/zirconocene generally offers higher catalyst activity than the other routes. Zirconocenes, Cp*_2_ZrCl_2_ (Cp* is a substituted or unsubstituted cyclopentadienyl ligand, Cp = η^5^-C_5_H_5_) are industrially important catalysts for the polymerization of α-olefins. (^*n*^BuCp)_2_ZrCl_2_ is the most representative metallocene compound, which is due to its stability, commercial availability with reasonable price, high activity in polymerization of ethylene, and can be used to synthesize supported metallocene catalysts.^4–6^ At present, metallocene catalysts are objects of increasing research interest particularly focusing on its stereochemical properties. Due to the formation of complexes, both the stereoselectivity of propylene and α-olefin polymerization activity are much higher. The steric hindrance of active center and the electronic modification due to π ligands are two very important factors to determine metallocene performance, particularly affecting the catalytic activity and the quality of the produced polymer.^7,8^

From the point of the coordination polymerization mechanism, the fewer electrons the core metal has, and the stronger the complexation between the metal and the olefin monomer is. Thus, the introduction of the electron-withdrawing substituent will accelerate the complexation step between the metal and the olefin monomer. On the other hand, the more electron the core metal as well as the stronger the feedback π bond has, the more weakened the double bond of the olefins is, so the introduction of electron-donating substituents can accelerate the double bond insertion rate. A polymerization reaction catalyzed by metallocene methylcations^9–15^ of group 4 is a complex multi-step process which essentially includes three kinds of key steps: initiation, propagation, and termination steps.^16^

The metallocene methyl cationic catalysts made from zirconium are known as zirconocene methyl cations and are typically produced by the reaction of Zr with neutral dichloromethane in the reaction with the cocatalyst,^17–19^ which is a Lewis acid capable of replacing a chlorine atom from one methyl group to produce an active substance, and the most notably is MAO.^20^ Several research groups have done systematic studies on the formation mechanism of active centers. Tritto used ^1^H NMR and ^13^C NMR to directly track the reaction process and confirm the structure of Cp_2_ZrCl_2_ and MAO.^21^ It was found that MAO has strongly alkylation effect. However, silica and MAO have structural heterogeneity. The silica includes tetrahedral SiO_4_ units, siloxane bridges (Si–O–Si)_*n*_ and silanols RSi–OH (as the surface ends). Silicone bridges can usually be 6 to 10 member rings, in addition, silanols can be geminal, vicinal and isolated.^22^ On the other hand, the cage structure of the MAO maintains the dynamic equilibrium between trimethylaluminum and the oligomers of MAO (–CH_3_OAl–)_*n*_.^23^ Thus, silica and MAO are potential sources of catalyst activity center multiplicity.

**Fig. 1:**
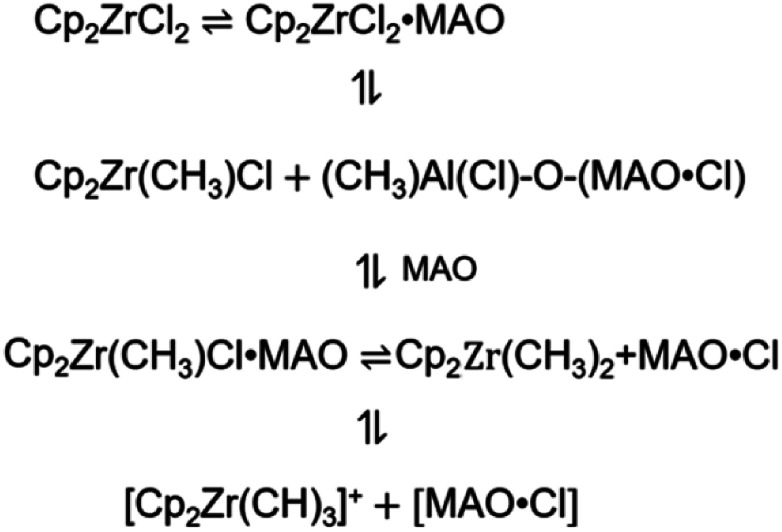
Formation of metallocene active center.

The metallocene compound reacts with MAO to form a cationic active site. Fig. 1 shows the main formation process of the active center. Different theoretical approaches have been used to study the reaction properties of these active sites. For the organic metal molecules in which the electron configuration of d(f) electrons is necessary, the complexity of orbital degeneracy and the change due to the presence of the ligand field of the molecule complicate even the theoretical analysis of the ground state. As a result, *ab initio* calculations based on density functional theory (DFT) are usually not available for experimental observations of ground state electronic configurations. In addition, the correlation effect of organometallic molecules may become important,^24,25^ further complicating the analysis. Although the reaction with a lower computed barrier height is usually the preferred pathway, rate constant computation is also required for a proper prediction of the preferred reaction pathway.

**Fig. 2:**
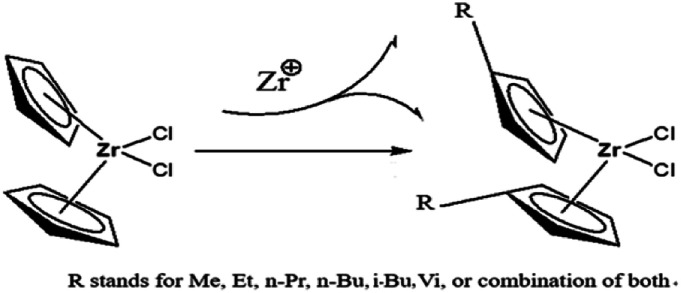
Schematic of introducing different substituent in the Cp ring.

In the paper, Cp_2_ZrCl_2_ was used as the basic metallocene compound, and different substituents were introduced into Cp to form new metallocene compounds (Fig. 2). The central metal atom charge density and band gap of the compound vary with the substituents. As a member of the metallocene compounds, the (^*n*^BuCp)_2_ZrCl_2_ compound appears to be more specific in charge density and band gap. For this reason, the charge density and phase change caused by *n*-butyl are calculated by DFT. The introduction of *n*-butyl also inevitably causes the change of polymerization mechanism between catalyst and olefin. The transition states of the reaction of the (^*n*^BuCp)_2_ZrCl_2_ compound with olefin were optimized by DFT, and the polymerization reaction mechanism and the structure of the final product of the catalyst and olefin were confirmed by combining the slurry polymerization experiment and the analysis of the test results.

## 2. Experimental

### 2.1 Chemicals and materials

All metallocene compounds were purchased from Alfa Aesar, a Johnson Matthey Company, and purity is 97%. MAO was purchased from Albemarle Corporation and the molar concentration is 1.0 mol L^−1^. *N*-Hexane and toluene were purchased from Lanzhou Petrochemical Company, the water content in which was removed using metal sodium. Silica gel was purchased from Grace Company in the United States for high temperature activation during catalyst loading experiments.

### 2.2 Catalyst loading and polymerization experiments

5.0 g silica gel carrier was added to the reactor, and 10% solution of MAO toluene was added to keep the aluminum content at about 10%, and the reaction was carried out at 0 to−20 °C for 2–4 hours. The compound was added to the reactor and the zirconium content was maintained at 0.2–0.4%. The Al/Zr molar ratio in the reaction solution was 200 : 1. The temperature of the reaction solution was heated to 40–70 °C, and the reaction was carried out at a constant temperature for 3–6 hours. After the reaction, the temperature was gradually lowered to room temperature, and then the treated toluene solution and the hexane solution were washed several times, and the catalyst was dried by vacuum filtration. The catalyst was then sealed with an inert gas for the next polymerization reaction.

A slurry polymerization tank (10 L) was charged with 3.4 L of hexane solvent while adding 10 mL of triethylaluminum (TEA) at a concentration of 1.0 mol L^−1^. With stirring turned on, 0.3 mg to 0.8 mg of catalyst was added after 15 min. The reaction temperature was raised to 83 °C and ethylene pressure was added to 1.2 MPa. After 1 hour, the reaction was allowed to be cooled to room temperature. The reactor was opened and the polymer was removed. After drying, the catalyst activity was calculated by weighing.

### 2.3 Calculation details

All calculations were carried out using the Dmol^3^ software in the Materials Studio package.^26,27^ A generalized gradient approximation (GGA) functional consisting of Becke's exchange and correlation expression proposed by Perdew, Burke, and Ernzerhof,^28^ localized double numerical basis sets with polarization functions (DND). In the SCF iterative calculation of the energy of the structures, orbital (HOMO/LUMO), frequency and population analysis was selected as properties parameter. The transition states (TS) were found by using the generalized linear synchronous and quadratic (LST/QST) proposed by Govind from the original methods of Halgren and Lipscomb.^29–32^ For the TS calculations, the LST was first maximized and then energy minimization was performed in the direction of conjugate with the relevant reaction path. The TS approximation found in this method was used to find the secondary synchronization transmission (QST) maximization and perform another conjugate gradient minimization. The cycle repeats until a minimum point was located. The cycle repeats until a minimum point was located. The convergence criterion for transition state calculations used a tolerance for all rms (root mean square) force values per atom as 0.25 eV Å^−1^. The LST/QST methods using the same convergence criteria were used in searching for transition states (TS). Calculating the possible vibration frequencies of the TS was identified, showing that each discovered TS had only one imaginary frequency.

## 3. Results and discussion

### 3.1 Calculation of population analysis

**Fig. 3:**
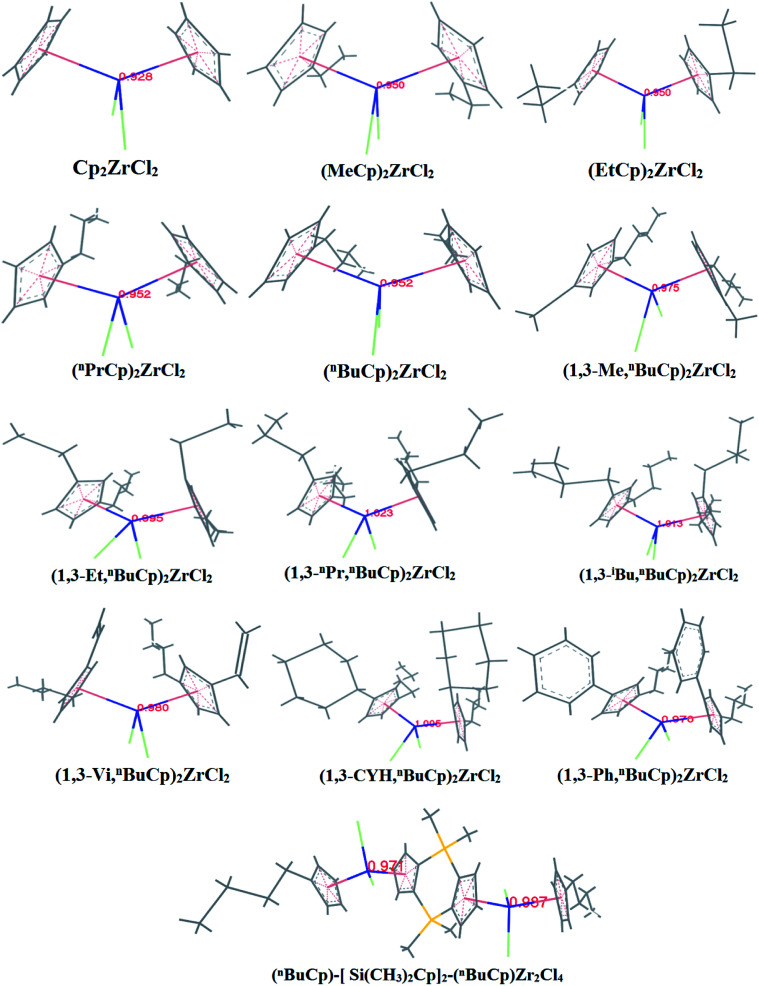
Charge density of zirconium atoms in metallocene compound.

**Fig. 4:**
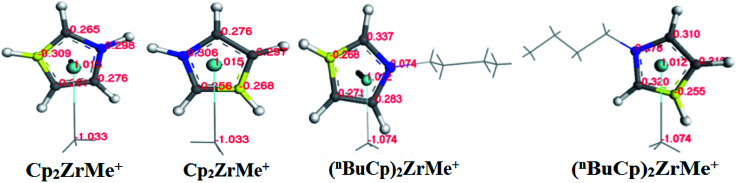
Distribution map of charge density of different atoms on the structure of two compounds (the blue carbon atom represents C1/C6, the yellow carbon atom represents C3/C8, the gray carbon atom represents C2/C4/C5 and C7/C9/C10).

**Fig. 5:**
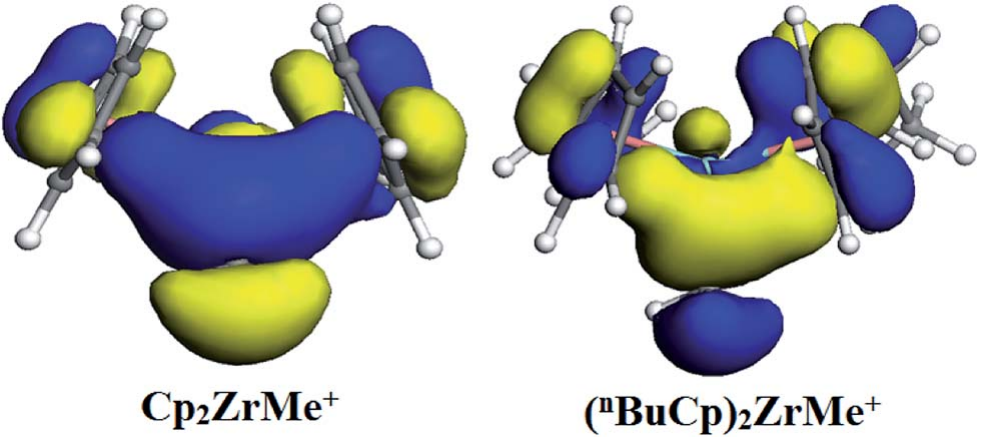
HOMO distribution of two compounds.

With the increases of the molecular weight of the alkyl substituent, the charge density of the central metal atom also increases (Table 1 and Fig. 3), indicating that the alkyl substituent exhibits an electron-donating effect. Of course, olefin and aromatic substituent exhibit electron-donating effects as well. Methylaluminoxane (MAO) reacts with the metallocene to form the cationic active center, where the charge density of the central metal atom is greatly improved (Table 2). Cationic active center can be coordinated with the olefin monomer to perform a polymerization reaction, at this time the central metal atomic charge density and catalyst activity are closely related. With the introduction of different alkyl groups on the Cp ring, the charge density of the central metal atoms increases in different degrees. However, when *n*-butyl is introduced, the charge density of the central metal atoms is reduced (Table 2). The introduction of *n*-butyl leads to an increase in the electron density near the atom (Table 3 and Fig. 4), exhibiting a strong electron-donating effect, and the charge density of the central metal atoms should increase; however, the results show a decrease in the charge density of the central metal atom (Table 3). For the central metal atom, it is shown that the substituent generally exhibits electron-withdrawing effects. The main reason for this contrast is the effect of Me–C. The HOMO/LUMO calculations of the two compounds show that the electronic state of Me–C present on the two compound structures is different (Fig. 5). The results are shown in. The positive and negative phases of the wave function produce by the methyl group in the structure of the two compounds are changed. Negative phase methyl groups lead to a significant reduction in the charge density of metal atoms in (^*n*^BuCp)_2_ZrMe^+^ compound.

**Table 1:** Calculation results of charge density of metal atoms in center

Compounds	Cp_2_ZrCl_2_	(MeCp)_2_ZrCl_2_	(EtCp)_2_ZrCl_2_	(^*n*^PrCp)_2_ZrCl_2_	(^*n*^BuCp)_2_ZrCl_2_	(1,3-Me,^*n*^BuCp)_2_ZrCl_2_	(1,3-Et,^*n*^BuCp)_2_ZrCl_2_	(1,3-^*n*^Pr,^*n*^BuCp)_2_ZrCl_2_	(1,3-^i^Bu,^*n*^BuCp)_2_ZrCl_2_	(1,3-Vi,^*n*^BuCp)_2_ZrCl_2_	(1,3-CYH,^*n*^BuCp)_2_ZrCl_2_	(1,3-Ph,^*n*^BuCp)_2_ZrCl_2_	(^*n*^BuCp)-[Si(CH_3_)_2_Cp]_2_-(^*n*^BuCp)Zr_2_Cl_4_	
Zr-charge	0.928	0.950	0.950	0.952	0.952	0.975	0.995	1.023	1.013	0.980	1.005	0.970	0.971/0.981	

**Table 2:** Calculation results of charge density of metal atoms after alkylation

Compounds	Cp_2_ZrMe^+^	(MeCp)_2_ZrMe^+^	(EtCp)_2_ZrMe^+^	(^*n*^PrCp)_2_ZrMe^+^	(^*n*^BuCp)_2_ZrMe^+^	(1,3-Me,^*n*^BuCp)_2_ZrMe^+^	(1,3-Et,^*n*^BuCp)_2_ZrMe^+^	(1,3-^*n*^Pr,^*n*^BuCp)_2_ZrMe^+^	(1,3-^i^Bu,^*n*^BuCp)_2_ZrMe^+^	(1,3-Vi,^*n*^BuCp)_2_ZrMe^+^	(1,3-CYH,^*n*^BuCp)_2_ZrMe^+^	(1,3-Ph,^*n*^BuCp)_2_ZrMe^+^	(^*n*^BuCp)-[Si(CH_3_)_2_Cp]_2_-(^*n*^BuCp)Zr_2_Me_2_^+^
Zr-charge	1.015	1.066	1.057	1.060	1.012	1.031	1.121	1.171	1.054	1.060	1.140	1.132	1.137/1.104

**Table 3:** Different atomic charge density distributions

Atoms	Cp_2_ZrMe^+^		(^*n*^BuCp)_2_ZrMe^+^	
C1/C6	−0.298	−0.306	0.074	0.078
C2/C7	−0.265	−0.256	−0.337	−0.320
C3/C8	−0.309	−0.268	−0.268	−0.255
C4/C9	−0.261	−0.297	−0.271	−0.310
C5/C10	−0.276	−0.276	−0.283	−0.310
Me–C	−1.033		−1.074	
Zr	1.015		1.012	

### 3.2 Calculations of HOMO/LUMO

**Fig. 6:**
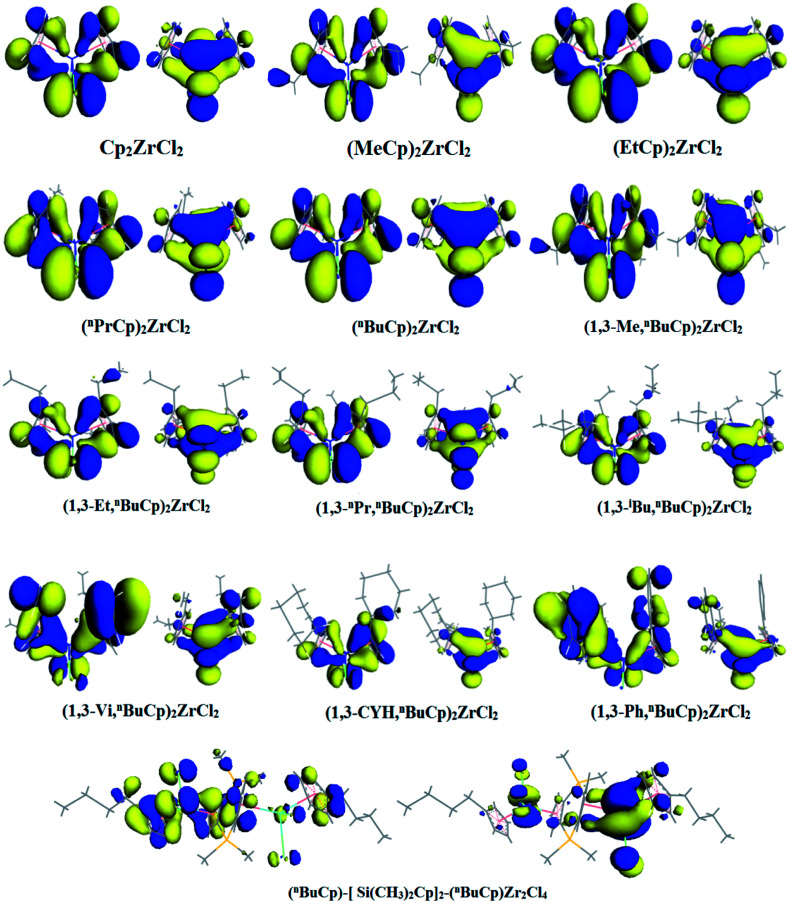
HOMO/LUMO distribution in different metallocene compounds.

When the different functional groups were introduced the cyclopentadienyl group of the compound, the effects were mainly electron effect and steric hindrance.^33^ The overwhelming steric hindrance will prevent olefin insertion reaction, resulting in inactivation of catalyst. The bonding orbital of different metallocene compounds is calculated (Fig. 6), where the energy difference between HOMO and LUMO is defined as energy band gap, Δ*E* = *E*_LUMO_ − *E*_HOMO_. Different metallocene compounds exhibit different band gap values (Table 4). When an unsaturated hydrocarbon or aromatic substituent is introduced into the cyclopentadienyl group, the band gap of the formed metallocene compound is smaller than that of the alkyl substituent. Among the metallocene compounds formed by the alkyl substituent, the band gap formed by the *n*-butyl substituent is the smallest. According to the Frontier orbital theory, we can see that the molecule with smaller band gap will be excited more easily.

**Table 4:** Bond energy and band gap distribution of different metallocene compounds

Orbital/Ha	Cp_2_ZrCl_2_	(MeCp)_2_ZrCl_2_	(EtCp)_2_ZrCl_2_	(^*n*^PrCp)_2_ZrCl_2_	(^*n*^BuCp)_2_ZrCl_2_	(1,3-Me,^*n*^BuCp)_2_ZrCl_2_	(1,3-Et,^*n*^BuCp)_2_ZrCl_2_	(1,3-^*n*^Pr,^*n*^BuCp)_2_ZrCl_2_	(1,3-^i^Bu,^*n*^BuCp)_2_ZrCl_2_	(1,3-Vi,^*n*^BuCp)_2_ZrCl_2_	(1,3-CYH,^*n*^BuCp)_2_ZrCl_2_	(1,3-Ph,^*n*^BuCp)_2_ZrCl_2_	(^*n*^BuCp)-[Si(CH_3_)_2_Cp]_2_-(^*n*^BuCp)Zr_2_Cl_4_
*E*_HOMO_	−0.2227	−0.2166	−0.2162	−0.2140	−0.2129	−0.2100	−0.2088	−0.2090	−0.2080	−0.2034	−0.2071	−0.2038	−0.2114
*E*_LUMO_	−0.1075	−0.1029	−0.1027	−0.1056	−0.1060	−0.0979	−0.0983	−0.0983	−0.0988	−0.1044	−0.0976	−0.1045	−0.1044
Δ*E*	0.1152	0.1137	0.1135	0.1084	0.1069	0.1121	0.1105	0.1107	0.1092	0.0990	0.1095	0.0993	0.1070

The metallocene underwent an alkylation reaction and became the active center of the electron deficient. The olefin monomer was first coordinated at the cationic active site and then inserted into the central metal-alkyl bond to complete the first step chain growth. The band gap of the active center formed by the alkylated metallocene compounds was calculated by DFT (Table 5). Comparing the data in Tables 4 and 5 we find that the band gap becomes narrower after these metallocene compounds are alkylated, indicating that the compound becomes more active, which is the role of MAO in the metallocene catalyst. The data in the Table 5 show that the band gap of the metallocene compound formed by the unsaturated hydrocarbon or the aromatic substituent is smaller relative to the alkyl substituent. However, the band gap of the metallocene compound formed by the *n*-butyl in the alkyl substituent appears to have a maximum value, which is in sharp contrast to the band gap formed before alkylation, following the metallocene compound by isobutyl and *n*-butyl, methyl and *n*-butyl as well.

**Table 5:** Bond energy and band gap distribution of different metallocene compounds after alkylation

Orbital/Ha	Cp_2_ZrMe^+^	(MeCp)_2_ZrMe^+^	(EtCp)_2_ZrMe^+^	(^*n*^PrCp)_2_ZrMe^+^	(^*n*^BuCp)_2_ZrMe^+^	(1,3-Me,^*n*^BuCp)_2_ZrMe^+^	(1,3-Et,^*n*^BuCp)_2_ZrMe^+^	(1,3-^*n*^Pr,^*n*^BuCp)_2_ZrMe^+^	(1,3-^i^Bu,^*n*^BuCp)_2_ZrMe^+^	(1,3-Vi,^*n*^BuCp)_2_ZrMe^+^	(1,3-CYH,^*n*^BuCp)_2_ZrMe^+^	(1,3-Ph,^*n*^BuCp)_2_ZrMe^+^	(^*n*^BuCp)-[Si(CH_3_)_2_Cp]_2_-(^*n*^BuCp)Zr_2_Me_2_^+^
*E*_HOMO_	−0.3803	−0.3645	−0.3622	−0.3562	−0.3535	−0.3388	−0.3427	−0.3359	−0.3493	−0.3275	−0.3314	−0.3155	−0.4148
*E*_LUMO_	−0.2837	−0.2692	−0.2655	−0.2672	−0.2488	−0.2396	−0.2540	−0.2538	−0.2455	−0.2568	−0.2482	−0.2542	−0.3322
Δ*E*	0.0966	0.0953	0.0967	0.0890	0.1047	0.0992	0.0887	0.0821	0.1038	0.0707	0.0832	0.0613	0.0826

### 3.3 Slurry polymerization of supported metallocene catalysts

The catalyst evaluation experiments were conducted by selecting the reaction temperature of 83 °C and 1.2 MPa in combination with the existing slurry polymerization process conditions. The ethylene slurry polymerization activity of catalysts formed from different metallocene compounds was calculated (Table 6).

**Table 6:** Metallocene catalyst slurry polymerization activity (83 °C, 1.2 MPa)^a^

Performance	Cp_2_ ZrCl_2_	(MeCp)_2_ZrCl_2_	(EtCp)_2_ZrCl_2_	(^*n*^PrCp)_2_ZrCl_2_	(^*n*^BuCp)_2_ZrCl_2_	(1,3-Me,^*n*^BuCp)_2_ZrCl_2_	(1,3-Et,^*n*^BuCp)_2_ZrCl_2_	(1,3-^*n*^Pr,^*n*^BuCp)_2_ZrCl_2_	(1,3-^i^Bu,^*n*^BuCp)_2_ZrCl_2_	(1,3-Vi,^*n*^BuCp)_2_ZrCl_2_	(1,3-CYH,^*n*^BuCp)_2_ZrCl_2_	(1,3-Ph,^*n*^BuCp)_2_ZrCl_2_	(^*n*^BuCp)-[Si(CH_3_)_2_Cp]_2_-(^*n*^BuCp)Zr_2_Cl_4_	HP-100
Polyethylene/g	590	572	638	446	1302	1080	340	292	1098	—	318	—	301	960
Activation/[kg (mol h)^−1^ × 10^4^]	5.40	5.22	5.82	4.07	11.88	9.86	3.10	2.66	10.02	—	2.90	—	2.75	8.77

a Where “—” represents the catalyst reaction inactive.

The catalyst activity by the (^*n*^BuCp)_2_ZrCl_2_ compound formed is the highest, followed by (1,3-^i^Bu,^*n*^BuCp)_2_ZrCl_2_ and (1,3-Me,^*n*^BuCp)_2_ZrCl_2_ compound, other compounds form a catalyst with low activity and are difficult to apply to industrial production. Compared Table 4 with Table 6, the results show that the higher the band gap of the metallocene compound is, the higher the polymerization activity will be. However, for the three compounds(1,3-Vi,^*n*^BuCp)_2_ZrCl_2_, (1,3-Ph,^*n*^BuCp)_2_ZrCl_2_ and (^*n*^BuCp)-[Si(CH_3_)_2_Cp]_2_-(^*n*^BuCp)Zr_2_Cl_4_, the olefin polymerization activity is reduced due to the steric hindrance. Comparison of Tables 5 and 6 shows that the larger the band gap formed by cations, the higher the activity of the formed catalyst. The Frontier orbit theory does not seem to be in use for cationic active centers. This is the characteristic of high-activity metallocene catalysts.

**Fig. 7:**
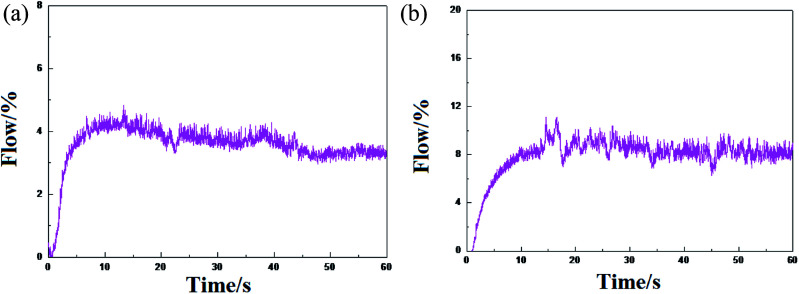
Trends in ethylene flow over time: (a) represents the metallocene catalyst olefin polymerization trend formed by the (^*n*^BuCp)_2_ZrCl_2_ compound, and (b) represents the tendency of the industrial metallocene catalyst HP-100 olefin polymerization.

The ethylene flow rate can reflect the high and low activity of the catalysts and the stable performance of the release activity. The difference in the polymerization activity of the catalyst HP-100 and formed by (^*n*^BuCp)_2_ZrCl_2_ compound is not great, however, there is a certain difference in the stability of the activity release (Fig. 7).

The metallocene catalyst formed by the (^*n*^BuCp)_2_ZrCl_2_ compound first starts to be initiated, the catalyst activity reaches the highest level, and then the activity slightly decreases, finally, the activity tends to be stable. However, HP-100 catalyst activity is apparently softer. There are many factors that leading to the decay of the catalyst activity. As far as the polymerization itself is concerned, nothing less than the increase of the polymerization reaction barrier and the generation of hydrogen in the process. Metallocene catalysts have a very good sensitivity to hydrogen; a small amount of hydrogen contributes to the increase of catalyst activity, but the presence of high concentration hydrogen will cause the catalyst to lose its activity.

The amount of polymer formed by polymerization of the catalysts at different reaction temperatures and its activity were verified by slurry polymerization experiments (Table 7).

**Table 7:** Polymerization activity of catalysts at different temperatures

Temperature/°C	Catalyst for the formation of (^*n*^BuCp)_2_ZrCl_2_		HP-100	
	Polymer/g	Catalyst activity/kg (mol h)^−1^ × 10^4^	Polymer/g	Catalyst activity/kg (mol h)^−1^ × 10^4^
40	141.8	1.75	50.27	0.62
50	264.7	2.94	90.02	1.00
60	370.6	3.94	125.4	1.33
70	903.4	8.24	320.8	2.93
80	1266.5	13.34	450.7	4.75
90	487.2	6.42	167.3	2.21

**Fig. 8:**
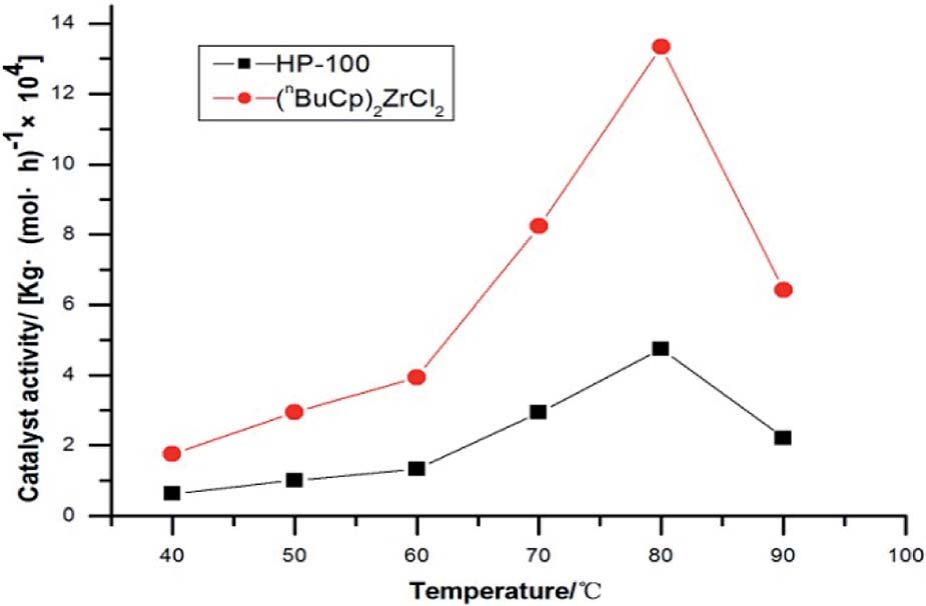
Catalyst activity with temperatures trend chart.

The activity of metallocene catalyst for slurry polymerization increases with the increases of reaction temperature. The polymerization activity of the two catalysts is the maximum when the reaction temperature reaches 80 °C or so. The polymerization activity of metallocene catalysts tends to decrease, as the reaction temperature continues to increase. The experimental results show that when the reaction temperature reaches 90 °C, the polymerization activity of the catalyst is greatly reduced, and the high temperature leads to the deactivation of the metallocene catalyst. The metallocene catalyst formed by the (^*n*^BuCp)_2_ZrCl_2_ compound is highly sensitive to temperature, while the sensitivity of HP-100 catalyst to temperature is somewhat alleviated (Fig. 8).

### 3.4 Transition state (TS) calculation of (^*n*^BuCp)_2_ZrCl_2_

**Fig. 9:**
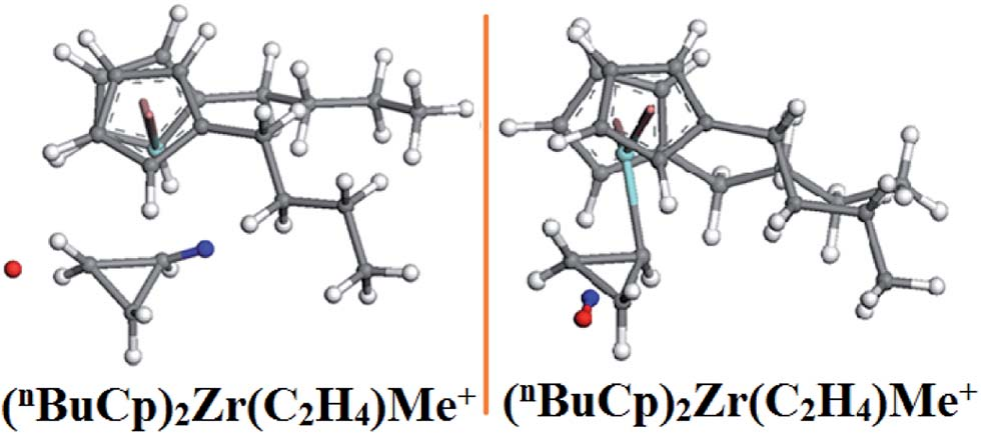
Transition structure before and after optimization.

The hydrogen content in the recycle gas is increased with the progress of the reaction without addition of hydrogen when the metallocene catalyst is subjected to a gas-phase fluidized-bed polymerization experiment, which indicates that the metallocene olefin releases a certain amount of hydrogen during the polymerization reaction. How is hydrogen produced? More relies on computational simulations to search for polymer transition states to explain the hydrogen release mechanism. The transition states (Fig. 9) during the reaction were simulated by DMol^3^ and the vibration was analyzed (Table 8).

**Table 8:** Vibration state before and after optimization of transition state

Vibration before optimization		Optimized vibration	
Frequency (cm^−1^)	Intensity (km mol^−1^)	Frequency (cm^−1^)	Intensity (km mol^−1^)
−675.69	34.47	−5075.87	51.40
−198.26	36.81	52.42	1.79
−114.51	0.30	70.06	0.30
−11.71	0.39	89.38	0.09
15.67	1.33	100.07	2.61
33.67	1.11	115.61	0.63
36.22	1.56	128.55	1.32
48.00	6.23	143.22	0.45
56.90	0.15	146.55	1.93
73.25	1.23	152.44	2.29

A dehydrogenation reaction occurs in the optimized structure (Fig. 9) of (^*n*^BuCp)_2_Zr(C_2_H_4_)Me^+^, the combination of hydride ion and hydrogen on the α-C of the growing chain results in the dehydrogenation reaction. There are multiple imaginary frequencies in the vibration analysis data, and the transition state needs to be optimized to obtain the corresponding unique imaginary frequency (Table 8). The transition state corresponds to the only imaginary frequency.

**Fig. 10:**
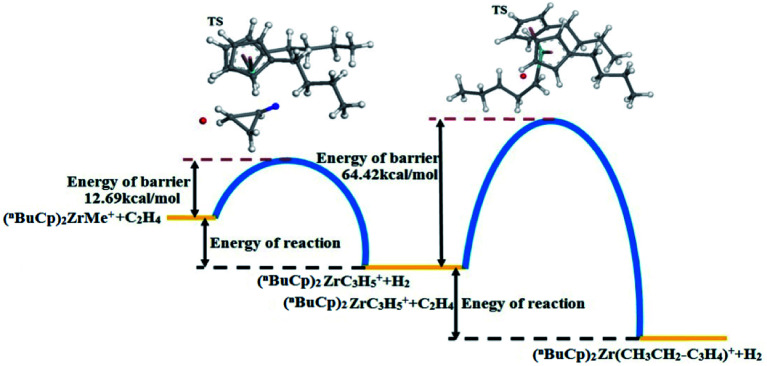
Reaction barrier diagram by the (^*n*^BuCp)_2_ZrMe^+^ compound.

The energy changes resulting from transition states were calculated (Table 9). The transition state energy generated by the (^*n*^BuCp)_2_ZrMe^+^ with olefins is −2735664.88 kcal mol^−1^ (*E*_LST_) and the energy of barrier is 83.01 kcal mol^−1^ during search for the transition states (Table 9). The optimized transition state energy is −2735735.20 kcal mol^−1^ (*E*_Opt_), the energy of transition state has a difference of −70.32 kcal mol^−1^ (*E*_Opt_ − *E*_LST_). Therefore, the optimized energy of barrier is approximately 12.69 kcal mol^−1^. Similarly, the transitional state barrier generated by secondary polymerization is 64.42 kcal mol^−1^. The optimization of the transition state structure makes the value of the reaction barrier energy correct (Table 10). The olefin polymerization process of the metallocene catalyst can be simulated through the modification of the transition state reaction barrier (Fig. 10).

**Table 9:** The energy generated by transition states

Energy/kcal mol^−1^	(^*n*^BuCp)_2_Zr(C_2_H_4_)Me^+^	(^*n*^BuCp)_2_Zr(C_2_H_4_)_2_Me^+^
Energy of reactant	−2 735 747.82	−2 786 154.25
Energy of product	−2 735 771.35	−2 786 167.54
Energy of transition state	−2 735 664.88	−2 786 037.88
Location of transition state	0.64	0.52
Energy of reaction	−23.467	−13.29
Energy of barrier	83.01	116.38

**Table 10:** The barrier to reaction before and after optimization

Energy of barrier/kcal mol^−1^	(^*n*^BuCp)_2_Zr(C_2_H_4_)Me^+^	(^*n*^BuCp)_2_Zr(C_2_H_4_)_2_Me^+^
TS search	83.01	116.38
TS optimization	12.69	64.42

The metallocene catalyst formed by the (^*n*^BuCp)_2_ZrMe^+^ compound in the polymerization of olefins shows that the first reaction barrier is lower than the secondary polymerization barrier potential. Of course, this is related to the formation of the metallocene catalyst in the polymerization process to release hydrogen. The micro-content of hydrogen contributes to the improvement of the catalytic activity of the metallocene catalyst, but the enrichment of hydrogen will cause the metallocene catalyst to lose its activity. On the other hand, transition state forms a Zr–cyclopropyl bond, and to allow the next monomer to be coordinated to the Zr, the Zr–cyclopropyl group must be rotated, and this rearrangement requires a certain amount of energy, the barrier is even higher than the barrier of the monomer insertion chain and becomes the main barrier in the whole chain growth process.

**Fig. 11:**
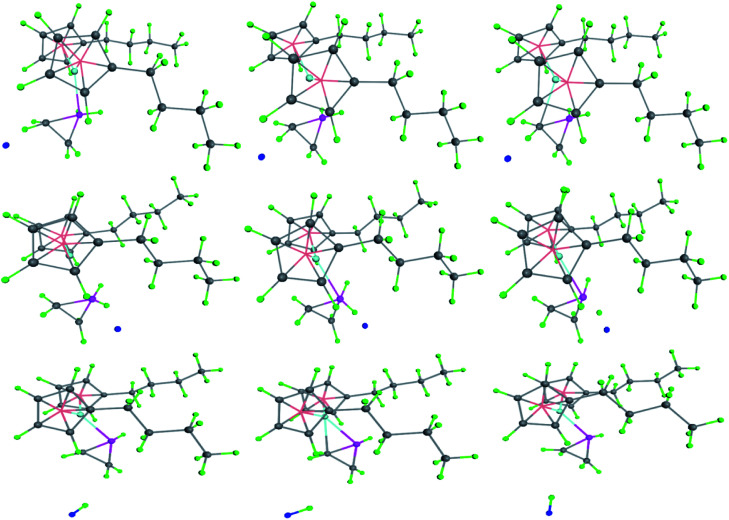
Hydrogen generation mechanism in transition state.

As for chain growth models of metallocene catalyzed olefin polymerization, there are 4 kinds of hydrogen production mechanisms. The commonly accepted model was that the hydrogen on the α-C of the growing chain interacted with the transition metal ion as electron-donor, known as the α-agostic effect.^34^ It can be seen from the figure that the α-agostic effect in the transition state resulting from the polymerization of the compound with the olefin monomer occurs twice, resulting in the production of hydrogen (Fig. 11). This is a typical ground state and transition state α-agostic reaction mechanism. It can be seen from the transition state reaction route that small molecule such as cyclopropane is produced during the polymerization of the (^*n*^BuCp)_2_ZrCl_2_ compound and the olefin. The cyclopropyl group as a ligand is never opened during the entire transition state, such as *n*-propyl or i-propyl structure. The structure of transition state is determined by the size of the band gap. It can also be seen from the transition state reaction path map that α-C and β-C of the growth chain produce competitive reactions, and eventually α-C occupies the leading dominance of the reaction, which is the origin of α-agostic effect.

### 3.5 Hydrogen content test

**Fig. 12:**
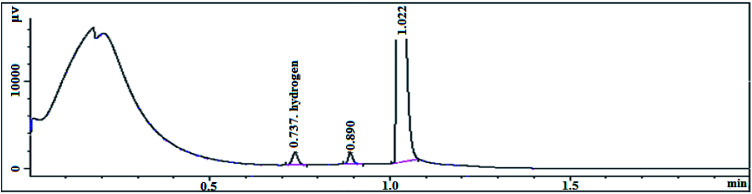
Chromatogram test chart.

The transition state produced by the polymerization of the compound with the olefin monomer indicates that the metallocene formed by the compound inevitably releases a large amount of hydrogen during slurry polymerization (Table 11). The on-line gas chromatograph is used to analyze and test the polymerization gas (Fig. 12).

**Table 11:** The result of hydrogen content test

Signal	Retention time/min	Area/(mAu × s)	Amt/area	Amount/ppm	Name
1	0.737	1527.93229	0.00002	235.2	Hydrogen

The reaction gas after the end of the polymerization reaction was slowly cooled to room temperature, using a sampling bag to collect gas for testing. It can be seen from the chromatographic peaks that the characteristic peak of hydrogen appears at 0.737 and the hydrogen content is 235.2 ppm. The metallocene catalyst formed by the (^*n*^BuCp)_2_ZrCl_2_ compound produces such large amount of hydrogen in the polymerization of the olefin that the melt index of the polymerization product is hard to be lower than 1.0 g/10 min, resulting in a limited commercial use. Therefore, looking for a metallocene catalyst with higher activity and lower hydrogen release is the next step.

## 4. Conclusion

In the case of metallocene compounds, the charge density of central metal atom increases with the introduction of substituent on the cyclopentadienyl groups, including alkyl groups, alkene groups, cycloalkyl groups, and aromatic groups. This shows that these functional groups exhibit electron-withdrawing effects. As these metallocene compounds react with MAO to form cationic active sites, the charge density of the central metal atom generally shows an increasing tendency; this is where MAO plays an important role in metallocene catalysts. The metallocene catalyst which forms the active center of the cation is subjected to the olefin slurry polymerization experiment under the same conditions and find that the smaller the charge density of central metal atom is, the higher the activity of the catalyst will be. The Frontier orbital theory suggests that the smaller the band gap is, the more easily the molecule will be excited. The results of DFT calculations show that the band gap of the metallocene compounds decreases after alkylation.

The (^*n*^BuCp)_2_ZrCl_2_ compound exhibits a more specific performance in both calculations, which is strongly related to the introduction of *n*-butyl. When the (^*n*^BuCp)_2_ZrCl_2_ compound is interacted with olefin, the barrier of the first reaction is significantly lower than that the secondary polymerization reaction, the reason for this situation is the reaction mechanism of compounds with olefins. The polymerization of the (^*n*^BuCp)_2_ZrCl_2_ compound and olefin is carried out according to the α-agostic reaction mechanism of the ground state and the transition state. Cyclopropane and hydrogen are generated during the reaction, and the final product exists as (^*n*^BuCp)_2_Zr(Cyclopropyl)^+^ structure, which is related to the band gap formed by itself.

The (^*n*^BuCp)_2_ZrMe^+^ shows particularity in the charge density of central metal atom and the band gap among the listed metallocene cation active centers. Compared with its olefin catalytic activity and reaction mechanism, it can be used as a basis to test the characteristics of metallocene compounds, which is the ultimate goal of writing this article.

## Conflicts of interest

There are no conflicts to declare.

## Supplementary Material
